# Nocturnal Hypoxia and Loss of Kidney Function

**DOI:** 10.1371/journal.pone.0019029

**Published:** 2011-04-29

**Authors:** Sofia B. Ahmed, Paul E. Ronksley, Brenda R. Hemmelgarn, Willis H. Tsai, Braden J. Manns, Marcello Tonelli, Scott W. Klarenbach, Rick Chin, Fiona M. Clement, Patrick J. Hanly

**Affiliations:** 1 Department of Medicine, University of Calgary, Calgary, Alberta, Canada; 2 Department of Community Health Sciences, University of Calgary, Calgary, Alberta, Canada; 3 Department of Medicine, University of Alberta, Edmonton, Alberta, Canada; 4 Alberta Kidney Disease Network, Calgary, Alberta, Canada; 5 Sleep Centre, Foothills Medical Centre, University of Calgary, Calgary, Alberta, Canada; Lerner Research Institute, Cleveland Clinic, United States of America

## Abstract

**Background:**

Although obstructive sleep apnea (OSA) is more common in patients with kidney disease, whether nocturnal hypoxia affects kidney function is unknown.

**Methods:**

We studied all adult subjects referred for diagnostic testing of sleep apnea between July 2005 and December 31 2007 who had serial measurement of their kidney function. Nocturnal hypoxia was defined as oxygen saturation (SaO2) below 90% for ≥12% of the nocturnal monitoring time. The primary outcome, accelerated loss of kidney function, was defined as a decline in estimated glomerular filtration rate (eGFR) ≥4 ml/min/1.73 m^2^ per year.

**Results:**

858 participants were included and followed for a mean study period of 2.1 years. Overall 374 (44%) had nocturnal hypoxia, and 49 (5.7%) had accelerated loss of kidney function. Compared to controls without hypoxia, patients with nocturnal hypoxia had a significant increase in the adjusted risk of accelerated kidney function loss (odds ratio (OR) 2.89, 95% confidence interval [CI] 1.25, 6.67).

**Conclusion:**

Nocturnal hypoxia was independently associated with an increased risk of accelerated kidney function loss. Further studies are required to determine whether treatment and correction of nocturnal hypoxia reduces loss of kidney function.

## Introduction

Chronic hypoxia and tubulointerstitial injury are common to all forms of kidney disease [1]. This has led to the “chronic hypoxia hypothesis” first proposed by Fine et al. [2] which emphasizes chronic ischemic damage in the tubulointerstitium as a final common pathway in end-stage kidney injury. Support for an association between hypoxia and development of progressive chronic kidney disease comes from numerous in-vitro and in-vivo studies of the effect of hypoxia on tubular and interstitial cells [3], while human physiology studies also demonstrate alterations in renal hemodynamics directly attributable to hypoxia [4], [5]. Although the Sleep Heart Health Study suggested an association between nocturnal hypoxia and risk of hypertension [6], reports on the effect of sleep disordered breathing on renal function are not consistent [7], [8], [9], [10], [11], [12], [13] and there are no studies examining loss of kidney function as the primary outcome.

Sleep diagnostic testing provides a unique opportunity to evaluate whether obstructive sleep apnea (OSA) and accompanying hypoxia are associated with accelerated loss of kidney function as patients undergo comprehensive assessment of their nocturnal oxygen saturation profile. The increasingly high prevalence of sleep-disordered breathing in the general community [14] coupled with the potential for nocturnal hypoxia to cause deterioration in kidney function [1], [2], [3] prompted our investigation of the relationship between nocturnal hypoxia and loss of kidney function in a cohort referred for evaluation of sleep apnea.

## Methods

### Objectives

The primary outcome was accelerated loss of kidney function, defined as a decrease in eGFR ≥4 mL/min/1.73 m^2^/year, which is more than double the anticipated normal rate of decline [15], [16]. The secondary outcome was the rate of loss of kidney function in mL/min/1.73 m^2^ per year.

### Participants

A cohort of all patients aged ≥18 years referred between July 2005 and December 2007 for diagnostic testing of sleep apnea to the Foothills Medical Centre (FMC) Sleep Centre or community respiratory care companies within the Calgary Health Region (population ∼1.3 million) were identified. Patients who had a previous diagnosis of sleep apnea or who had prior diagnostic testing (polysomnography or nocturnal cardio-pulmonary monitoring) were excluded.

### Description of Procedures or Investigations undertaken

Using the unique provincial health care number for each subject, this cohort of patients with diagnostic sleep testing was linked to the Alberta Kidney Disease network repository of laboratory data to determine out-patient serum creatinine measurements [17]. Given that sleep apnea is a chronic condition [18], we included outpatient creatinine measurements in the one year period *prior* to the sleep study for assessment of baseline kidney function. To be eligible for inclusion, participants required 2 or more outpatient creatinine measurements during the study period (i.e. from 1 year prior to their sleep test to the end of follow-up (December 31, 2007)) to enable assessment of serial kidney function. The first serum creatinine result in the study period was used to define baseline kidney function and subsequent measurements were used to determine rate of change in kidney function. Subjects were censored at death or study end. While all available serum creatinine measurements within the study period were used to derive the rate of loss, serum creatinine measurements associated with a hospital admission were excluded to reduce the risk that episodes of acute kidney injury would be classified as accelerated loss of kidney function [16]. Patients were also excluded if they were receiving renal replacement therapy (hemodialysis, peritoneal dialysis, or kidney transplant) at study enrolment [19].

### Measurement of Kidney Function and Definition of Outcomes

Estimated glomerular filtration rate (eGFR) was used to estimate kidney function using the abbreviated Modification of Diet in Renal Disease (MDRD) equation [20]. Although data for race were not available, misclassification of eGFR was expected to be minimal because less than 2% of the Alberta population is black [21]. Given concerns about the validity of the MDRD equation for subjects with higher levels of kidney function [22], patients with baseline eGFR values exceeding 90 mL/min/1.73 m^2^ were excluded.

### Study Variables and their Measurement

#### Sleep and Oxygenation Assessment

All subjects underwent attended polysomnography (PSG) in the sleep laboratory at Foothills Medical Centre or unattended nocturnal cardio-pulmonary monitoring at home (Remmers Sleep Recorder, SagaTech Electronics Inc., Calgary, Canada). PSG data were recorded by a computerized polysomnographic system (Sandman Elite Version 8.0, Nellcor Puritan Bennett (Melville) Ltd, Kanata, Ontario, Canada). This included a standardized montage: three channel electroencephalograms (C4/A1, C3/A2, O1/A2), bilateral electro-oculograms (EOG), submental electromyogram (EMG), bilateral leg EMGs, and electrocardiography (ECG). Airflow was measured using a nasal pressure transducer (Braebon Medical Corp, Ontario, Canada). Respiratory effort was assessed by inductance plethysmography (Respitrace Ambulatory Monitoring, Ardsley, New York, USA), and arterial oxygen saturation (SaO2) was recorded with a pulse oximeter (Biox 3740, Ohmeda, Boulder, Colorado, USA). The Remmers Sleep Recorder is an ambulatory monitor, which measures snoring, arterial oxygen saturation, respiratory airflow, EKG and body position. The Remmers Sleep Recorder has been reported to have excellent agreement, sensitivity and specificity with PSG [23], [24].

### Measures of exposure

#### Nocturnal hypoxia

Nocturnal hypoxia was defined as a SaO2 below 90% for ≥12% of the total nocturnal monitoring time. A similar metric of nocturnal hypoxia has been used in the Sleep Heart Health study [6].

#### Respiratory Disturbance Index

During PSG, the RDI was derived from manual scoring of the number of apneas and hypopneas per hour of sleep. Apnea was defined as a cessation of airflow for at least 10 s. Hypopnea was defined as an abnormal respiratory event lasting 10 s or more, with at least a 30% reduction in thoraco-abdominal movement or airflow compared with baseline and associated with at least a 4% oxygen desaturation [25]. During nocturnal cardio-pulmonary monitoring, the RDI was derived from automated analysis of the oximetry signal using a 4% desaturation threshold [24].

#### Respiratory Disturbance Index

The RDI is used to assess the presence and severity of sleep apnea [25]. During PSG, the RDI was derived from manual scoring of the number of apneas and hypopneas per hour of sleep. Apnea was defined as a cessation of airflow for at least 10 s. Hypopnea was defined as an abnormal respiratory event lasting 10 s or more, with at least a 30% reduction in thoraco-abdominal movement or airflow compared with baseline and associated with at least a 4% oxygen desaturation [25]. During nocturnal cardio-pulmonary monitoring with the Remmers Sleep Recorder, the RDI was derived from automated analysis of the oximetry signal using a 4% desaturation threshold. Our sleep centre has longstanding experience with this monitoring device [23]. It has been validated by comparison to attended PSG in the sleep laboratory which showed that the RDI derived by the Remmers Sleep Recorder was highly correlated with the RDI derived by PSG (r = 0.97) with a sensitivity and specificity of 98% and 88% respectively [24]. More recently, other investigators have validated its use in the management of patients with obstructive sleep apnea [26].

#### Obstructive Sleep Apnea (OSA)

OSA exposure was defined as recommended by the American Academy of Sleep Medicine [25]. Subjects were stratified by OSA severity based on two RDI cut-points: RDI≥15 events/hr and RDI≥30 events/hr (severe OSA).

### Measurement of Covariates

Baseline clinical and demographic information was collected for all participants at the time of sleep diagnostic testing including age, gender, body mass index (BMI), neck circumference and smoking status. Comorbidity was determined through the use of a questionnaire administered by trained personnel. Patients were asked to report the presence of specific comorbidities including hypertension, myocardial infarction, heart failure, cardiac arrhythmia, stroke, asthma, chronic obstructive pulmonary disease (COPD), diabetes and depression.

Validated algorithms using administrative data were employed to define diabetes [27], asthma [28], stroke [29], myocardial infarction [30], heart failure [31], and hypertension [32]. Using either self-report or administrative data, an enhanced measure of comorbidity was developed. This method has been shown to have face validity and provide clinically meaningful trends in the prevalence of comorbidity among this population [33].

### Ethics

The study was approved by the Conjoint Ethics Review Board at the University of Calgary. A waiver of consent was granted for this study, as the individual consent obtained from participants in the Sleep Cohort study allowed for monitoring use of health care resources, which included laboratory data.

### Statistical Methods

Baseline participant characteristics by presence of nocturnal hypoxia are presented as the mean ± standard deviation (SD) for normally distributed continuous variables and proportions for dichotomous variables. Differences in baseline characteristics between categories of nocturnal hypoxia were determined by chi-squared test or t-test. A mixed-effects model [15] was used to determine the rate of loss in eGFR in mL/min/1.73 m^2^ per year, with accelerated decline defined as eGFR loss ≥4 mL/min/1.73 m^2^ per year. The association between nocturnal hypoxia and accelerated loss of kidney function was assessed using multiple logistic regression. Initial univariate models were developed to identify significant predictors of accelerated loss of kidney function. Using these predictors, saturated multivariate models were constructed and subsequently reduced by backward elimination techniques. We removed highly insignificant variables, one at a time, and compared nested models by using the likelihood ratio test to determine if there was a significant difference between the models. The most parsimonious model prior to finding a significant difference between models was used. Subjects without nocturnal hypoxia formed the reference group in this analysis.

Similar models were developed to determine the association between measures of OSA and accelerated loss of kidney function. OSA was modeled as both a continuous and dichotomous variable. In the later models, varying RDI cut-points were used to define OSA severity (RDI≥15 events/hr and RDI≥30 events/hr).

In all analyses, the assumptions for logistic regression models were tested and met. All analyses were conducted using SAS (version 9.2, SAS Institute Inc., Cary, NC) or Stata (version 10.0; Stata Corp, College Station, TX) with 2-tailed significance levels of 0.05.

## Results

**Figure 1:**
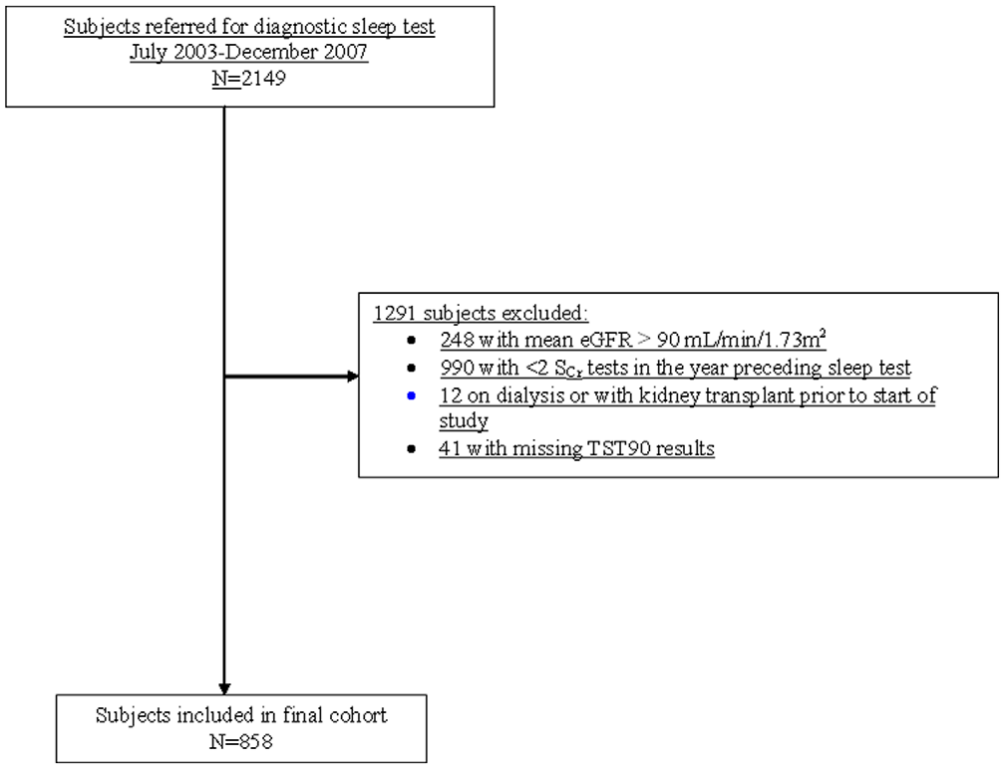
Formation of study cohort and criteria for exclusion. Abbreviations: eGFR, estimated glomerular filtration rate; S_Cr_, serum creatinine; TST90, total sleep time spent with oxygen saturation <90%.

During the study period, 2149 participants were referred for diagnostic testing of sleep apnea. As outlined in Figure 1, 1291 (60%) participants did not meet the inclusion criteria, resulting in a final study cohort of 858 subjects. Of the 858 participants, 696 (81%) underwent testing using the Remmers Sleep Recorder, and 162 (19%) underwent PSG. In general, participants with nocturnal hypoxia tended to be older, male, have a greater BMI and neck circumference and a lower baseline eGFR (Table 1). In addition to demonstrating a higher prevalence of severe OSA (RDI≥30 events/hr), participants with nocturnal hypoxia were also more likely to have hypertension, diabetes, and a history of cardiovascular disease compared to subjects without nocturnal hypoxia. Participants with nocturnal hypoxia were also more likely to be receiving angiotensin converting enzyme-inhibitor (ACE-I) or angiotensin receptor blockers (ARB) or cardiovascular medications (including acetylsalicylic acid, nitrates, calcium channel blockers, arrhythmia medications, and lipid-lowering agents) at the time of sleep assessment.

**Table 1:** Baseline subject characteristics, overall and by presence of nocturnal hypoxia*.

	*Overall*	*Nocturnal Hypoxia Absent*	*Nocturnal Hypoxia Present*	*p-value* †
	*n = 858*	*n = 484*	*n = 374*	
Age (years)	55.2 (12.4)	52.8 (12.5)	58.4 (11.6)	<0.001
Males (%)	54.9	51.7	59.1	0.03
Current Smoker (%)	12.4	11.0	14.2	0.2
Baseline eGFR (ml/min/1.73 m^2^)	70.8 (12.3)	72.3 (11.3)	68.8 (13.3)	<0.001
Baseline eGFR <60 ml/min/1.73 m^2^ (%)	18.0	13.2	24.1	<0.001
SaO2<90% (% nocturnal monitoring)	21.8 (27.5)	3.1 (3.27)	46.0 (26.1)	<0.001
RDI	22.9 (25.2)	12.3 (12.0)	36.5 (30.7)	<0.001
RDI≥15, %	46.2	26.7	71.4	<0.001
RDI≥30, %	24.6	8.3	45.7	<0.001
BMI (kg/m^2^)	32.8 (7.5)	30.6 (6.7)	35.7 (7.5)	<0.001
Neck Circumference (in)‡	15.9 (1.9)	15.3 (1.7)	16.7 (1.8)	<0.001
Co-morbidities (%)				
Hypertension	55.2	46.5	66.6	<0.001
Diabetes	19.6	13.6	27.3	<0.001
Depression	37.3	38.0	36.4	0.6
Asthma	23.7	21.1	27.0	0.04
COPD	7.5	4.6	11.2	<0.001
Myocardial Infarction	14.6	10.5	19.8	<0.001
Heart Failure	7.0	3.7	11.2	<0.001
Stroke	4.6	2.7	7.0	0.003
Medication Use, %				
No Medications	13.8	18.2	8.0	<0.001
ACEI/ARB	32.8	23.4	44.9	<0.001
Sedative/Hypnotics	11.7	11.4	12.0	0.8
Inhaled Steriods	7.8	5.2	11.2	<0.001
Cardiovascular Medications	37.9	29.3	48.9	<0.001

Abbreviations: eGFR, estimated glomerular filtration rate; RDI, respiratory disturbance index; BMI, body mass index; COPD, chronic obstructive pulmonary disease; ACEI, angiotensin converting enzyme inhibitor; ARB, angiotensin receptor blocker.

Cardiovascular Medications include acetylsalicylic acid, nitrates, calcium channel blockers, beta blockers, arrhythmia medications, and lipid lower medications.

*Results presented as mean (standard deviation) unless otherwise indicated.

† p-value for categorical variables based on a chi-square test of independence; p-value for continuous variables based on a 2-sample t-test for a difference, assuming equal variances.

‡ Neck Circumference: n = 683 (Overall), n = 381 (Nocturnal Hypoxia absent), n = 302 (Nocturnal Hypoxia present).

Of the 858 participants, 374 (44%) had nocturnal hypoxia and 49 (5.7%) were identified as having accelerated loss of kidney function. Over a mean (SD) follow-up of 2.1 (0.6) years, the loss of kidney function was 0.51 ml/min/1.73 m^2^/yr greater in participants with nocturnal hypoxia compared to participants without nocturnal hypoxia) (p<0.001).

The association between covariates and accelerated loss of kidney function are outlined in Table 2. As anticipated, age, presence of hypertension, presence of diabetes, and a history of cardiovascular disease were each associated with more accelerated loss of kidney function. Both continuous and categorical measures of OSA (as defined by RDI) were also independent predictors of accelerated loss of kidney function. Compared to patients without nocturnal hypoxia, those with nocturnal hypoxia experienced a greater than six-fold increase in the unadjusted risk of accelerated loss of kidney function (Table 3). After adjustment for RDI, age, body mass index, diabetes and heart failure, the risk was reduced slightly but remained nearly three-fold higher than in controls (OR: 2.89, 95% CI; 2.89, 1.25–6.67). Results were unaffected by further adjustment for ACE-I/ARB use.

**Table 2:** Univariate analysis for risk of accelerated loss of kidney function.

Covariate	Risk of Accelerated Loss of Kidney Function (OR (95% CI)
Male sex	1.44 (0.79, 2.62)
Current Smoker	1.20 (0.52, 2.73)
Age (per year)	1.06 (1.03, 1.09)
Body mass index (per kg/m^2^)	1.07 (1.03, 1.10)
Diabetes mellitus	10.30 (5.51, 19.24)
History of congestive heart failure	6.63 (3.34, 13.20)
Hypertension	6.29 (2.65, 14.93)
History of myocardial infarction	2.80 (1.48, 5.32)
History of stroke	2.59 (0.97, 6.95)
ACEI/ARB Use	2.93 (1.63, 5.26)
RDI (events/hr)	1.02 (1.01, 1.03)
OSA (≥15 events/hr)	2.09 (1.15, 3.81)
OSA (≥30 events/hr)	4.17 (2.32, 7.49)

Abbreviations: ACEI, angiotensin converting enzyme inhibitor; ARB, angiotensin receptor blocker; RDI, respiratory disturbance index; OSA, obstructive sleep apnea.

**Table 3:** Association between nocturnal hypoxia and risk of accelerated loss of kidney function*.

	Unadjusted Model OR (95% CI)	Multivariate adjusted model† OR (95% CI)	Multivariate adjusted model‡ OR (95% CI)
Nocturnal hypoxia	6.32 (3.03, 13.20)	3.38 (1.53, 7.45)	2.89 (1.25, 6.67)

Abbreviations: OR, odds ratio; CI, confidence interval.

*Reference group is subjects without nocturnal hypoxia.

† Adjusted for age, body mass index, diabetes and heart failure.

‡ Adjusted for respiratory disturbance index (RDI), age, body mass index, diabetes and heart failure.

In unadjusted logistic regression, severe OSA (RDI≥30 events/hr) was associated with an increased risk of accelerated loss of kidney function compared to patients with RDI<30 events/hr (OR: 4.16, 95% CI; 2.32, 7.49). However, this relationship was no longer significant after adjustment for age, BMI, diabetes, heart failure, and nocturnal hypoxia status (OR: 1.68, 95% CI; 0.85, 3.31). Similar results were obtained when RDI≥15 events/hr was used to define disease (OR: 0.71, 95% CI; 0.35, 1.46) or when RDI was modeled as a continuous variable (OR: 1.01, 95% CI; 1.00, 1.02).

## Discussion

Compared to subjects without nocturnal hypoxia, subjects with nocturnal hypoxia demonstrated an almost three-fold increase in the risk of accelerated loss of kidney function, even after adjustment for factors conventionally associated with loss of kidney function such as age, body mass index, diabetes and heart failure.

While previous studies have addressed the impact of hypoxia on urinary protein excretion [7], [8], [9], [10], [11], [12], [13], few have examined the effects of hypoxia on loss of kidney function. A large retrospective cross-sectional study of patients undergoing PSG for suspected sleep-disordered breathing demonstrated a significantly higher prevalence of chronic kidney disease (CKD) compared to that of age- and sex-matched controls [34], although the impact of hypoxia on the risk of developing and accelerating CKD was not addressed. A strong association between nocturnal hypoxia and risk of hypertension has also been demonstrated in a large cross-sectional community-based study [6], with subjects experiencing the greatest duration of sleep with an SaO2 below 90% having a 46% increased risk of hypertension. In our study the association between nocturnal hypoxia and accelerated loss of kidney function persisted even after adjustment for hypertension and use of angiotensin-converting enzyme inhibitors and angiotensin receptor blockers, suggesting that factors other than hypertension may be responsible.

There is increasing evidence that hypoxia has a direct effect on kidney function. Glomerulomegaly and focal segmental glomerulosclerosis have both been observed in human renal biopsies in patients with the sleep apnea syndrome [3], [13] and hypoxia is a common underlying mechanism for CKD progression through tubulointerstitial injury [1]. In animal studies, intermittent hypoxia causes a progressive increase in blood pressure, mediated in part through renal sympathetic nerve activity that acts to increase RAS activity [35]. In humans, polymorphisms in the angiotensin-converting enzyme (ACE) gene modulate susceptibility to hypertension in sleep apnea [36], and increased RAS activity is associated with glomerular hyperfiltration, a precursor to kidney disease. Nocturnal hypoxia predicts greater glomerular pressure in patients with chronic obstructive pulmonary disease [4]. and OSA has been shown to be independently associated with glomerular hyperfiltration [5]. Furthermore, treatment of OSA with continuous positive airway pressure resulted in a significant decrease in glomerular pressure [5], suggesting that reversal of hypoxia may improve kidney function, though this is clearly speculative. Interestingly, a negative association between the prevalence of CKD and end-stage kidney disease and altitude of residence has been shown [37], indicating that the renal response to hypoxia may differ depending on whether the exposure is chronic or intermittent.

Our study has many strengths. While previous studies have shown that sleep apnea is common in adults undergoing maintenance dialysis [38], [39], [40], less is known about the potential impact of sleep apnea and accompanying hypoxia on kidney function in patients with CKD [41]. To our knowledge, this is the first study to examine the association between nocturnal hypoxia and loss of kidney function. Furthermore, we were able to control for several comorbidities which can also contribute to a decline in kidney function. The size of the cohort and the fact that all patients were referred by primary care physicians increase the generalizability of our findings. Finally, we did not select patients based on the presence and severity of CKD which reduced the potential for biased recruitment of those at increased risk of a decline in kidney function.

### Limitations

Our study also has limitations. First, as in all observational studies, there is the potential for residual confounding. However, we were able to control for important clinical data that have been shown to impact loss of kidney function, including hypertension [27], use of ACE-I or ARBs [14] and use of non-steroidal anti-inflammatory drugs [28]. We do not have information on alcohol consumption in our study population. However, alcohol consumption does not appear to impact loss of kidney function [42], [43], [44]. Common practice dictates that renal hemodynamic parameters be indexed to body surface area (BSA) [45], yet it has been suggested that normalizing to BSA may be inappropriate in the obese [46], [47]. However. as we are measuring loss of kidney function *within* subjects rather than *between* subjects, the accuracy will be the same for all measurements.

Further, subjects were recruited from patients referred for diagnostic testing for sleep apnea, which raises the possibility of referral bias. However, all patients seen at community respiratory care companies as well as at the FMC Sleep Centre had diagnostic testing prior to consultation with a sleep physician. Our patient population was not selected by sleep physicians, but rather by their primary care physicians who suspected the presence of sleep apnea. Lastly, although the study is longitudinal the relatively short duration of follow-up does not exclude reverse causality, i.e. those with faster decline in kidney function develop nocturnal hypoxia.

In summary, we found that nocturnal hypoxia was associated with an increased risk of accelerated loss of kidney function in this population referred for diagnostic testing of sleep apnea, suggesting that surveillance of kidney function may be prudent in patients with sleep disordered breathing. Furthermore, it may be worthwhile to identify patients with CKD at risk for nocturnal hypoxia, particularly if their renal function is declining despite conventional management. Although it remains unclear whether correction of nocturnal hypoxia improves kidney function, we believe that this hypothesis merits further study.

## References

[1] Nangaku M (2006). Chronic hypoxia and tubulointerstitial injury: a final common pathway to end-stage renal failure.. J Am Soc Nephrol.

[2] Fine LG, Orphanides C, Norman JT (1998). Progressive renal disease: the chronic hypoxia hypothesis.. Kidney Int Suppl.

[3] Fine LG, Norman JT (2008). Chronic hypoxia as a mechanism of progression of chronic kidney diseases: from hypothesis to novel therapeutics.. Kidney Int.

[4] Bratel T, Ljungman S, Runold M, Stenvinkel P (2003). Renal function in hypoxaemic chronic obstructive pulmonary disease: effects of long-term oxygen treatment.. Respir Med.

[5] Kinebuchi S, Kazama JJ, Satoh M, Sakai K, Nakayama H (2004). Short-term use of continuous positive airway pressure ameliorates glomerular hyperfiltration in patients with obstructive sleep apnoea syndrome.. Clin Sci (Lond).

[6] Nieto FJ, Young TB, Lind BK, Shahar E, Samet JM (2000). Association of sleep-disordered breathing, sleep apnea, and hypertension in a large community-based study. Sleep Heart Health Study.. Jama.

[7] Bailey RR, Lynn KL, Burry AF, Drennan C (1989). Proteinuria, glomerulomegaly and focal glomerulosclerosis in a grossly obese man with obstructive sleep apnea syndrome.. Aust N Z J Med.

[8] Chaudhary BA, Sklar AH, Chaudhary TK, Kolbeck RC, Speir WA (1988). Sleep apnea, proteinuria, and nephrotic syndrome.. Sleep.

[9] Sklar AH, Chaudhary BA (1988). Reversible proteinuria in obstructive sleep apnea syndrome.. Arch Intern Med.

[10] Sklar AH, Chaudhary BA, Harp R (1989). Nocturnal urinary protein excretion rates in patients with sleep apnea.. Nephron.

[11] Faulx MD, Storfer-Isser A, Kirchner HL, Jenny NS, Tracy RP (2007). Obstructive sleep apnea is associated with increased urinary albumin excretion.. Sleep.

[12] Casserly LF, Chow N, Ali S, Gottlieb DJ, Epstein LJ (2001). Proteinuria in obstructive sleep apnea.. Kidney Int.

[13] Mello P, Franger M, Boujaoude Z, Adaimy M, Gelfand E (2004). Night and day proteinuria in patients with sleep apnea.. Am J Kidney Dis.

[14] Young T, Peppard PE, Gottlieb DJ (2002). Epidemiology of obstructive sleep apnea: a population health perspective.. Am J Respir Crit Care Med.

[15] Hemmelgarn BR, Zhang J, Manns BJ, Tonelli M, Larsen E (2006). Progression of kidney dysfunction in the community-dwelling elderly.. Kidney Int.

[16] Ahmed SB, Culleton BF, Tonelli M, Klarenbach SW, Macrae JM (2008). Oral estrogen therapy in postmenopausal women is associated with loss of kidney function.. Kidney Int.

[17] Hemmelgarn BR, Clement F, Manns BJ, Klarenbach S, James MT (2009). Overview of the Alberta Kidney Disease Network.. BMC Nephrol.

[18] Baumel MJ, Maislin G, Pack AI (1997). Population and occupational screening for obstructive sleep apnea: are we there yet?. Am J Respir Crit Care Med.

[19] Manns BJ, Mortis GP, Taub KJ, McLaughlin K, Donaldson C (2001). The Southern Alberta Renal Program database: a prototype for patient management and research initiatives.. Clin Invest Med.

[20] Levey AS, Greene T, Kusek JW, Beck GL (2000). MDRD Study Group. A simplified equation to predict glomerular filtration rate from serum creatinine (abstract). J Am Soc Nephrol.

[21] Statistics Canada

[22] Lin J, Knight EL, Hogan ML, Singh AK (2003). A comparison of prediction equations for estimating glomerular filtration rate in adults without kidney disease.. J Am Soc Nephrol.

[23] Issa FG, Morrison D, Hadjuk E, Iyer A, Feroah T (1993). Digital monitoring of sleep-disordered breathing using snoring sound and arterial oxygen saturation.. Am Rev Respir Dis.

[24] Vazquez JC, Tsai WH, Flemons WW, Masuda A, Brant R (2000). Automated analysis of digital oximetry in the diagnosis of obstructive sleep apnoea.. Thorax.

[25] Epstein LJ, Kristo D, Strollo PJ, Friedman N, Malhotra A (2009). Clinical guideline for the evaluation, management and long-term care of obstructive sleep apnea in adults.. J Clin Sleep Med.

[26] Mulgrew AT, Fox N, Ayas NT, Ryan CF (2007). Diagnosis and initial management of obstructive sleep apnea without polysomnography: a randomized validation study.. Ann Intern Med.

[27] Hux JE, Ivis F, Flintoft V, Bica A (2002). Diabetes in Ontario: determination of prevalence and incidence using a validated administrative data algorithm.. Diabetes Care.

[28] Huzel L, Roos LL, Anthonisen NR, Manfreda J (2002). Diagnosing asthma: the fit between survey and administrative database.. Can Respir J.

[29] Kokotailo RA, Hill MD (2005). Coding of stroke and stroke risk factors using international classification of diseases, revisions 9 and 10.. Stroke.

[30] Austin PC, Daly PA, Tu JV (2002). A multicenter study of the coding accuracy of hospital discharge administrative data for patients admitted to cardiac care units in Ontario.. Am Heart J.

[31] Lee DS, Donovan L, Austin PC, Gong Y, Liu PP (2005). Comparison of coding of heart failure and comorbidities in administrative and clinical data for use in outcomes research.. Med Care.

[32] Tu K, Campbell NR, Chen ZL, Cauch-Dudek KJ, McAlister FA (2007). Accuracy of administrative databases in identifying patients with hypertension.. Open Med.

[33] Ronksley PE, Tsai WH, Quan H, Faris P, Hemmelgarn BR (2009). Data enhancement for co-morbidity measurement among patients referred for sleep diagnostic testing: an observational study.. BMC Med Res Methodol.

[34] Iseki K, Tohyama K, Matsumoto T, Nakamura H (2008). High Prevalence of chronic kidney disease among patients with sleep related breathing disorder (SRBD).. Hypertens Res.

[35] Fletcher EC, Bao G, Li R (1999). Renin activity and blood pressure in response to chronic episodic hypoxia.. Hypertension.

[36] Bostrom KB, Hedner J, Melander O, Grote L, Gullberg B (2007). Interaction between the angiotensin-converting enzyme gene insertion/deletion polymorphism and obstructive sleep apnoea as a mechanism for hypertension.. J Hypertens.

[37] Ghahramani N, Ahmed F, Al-Laham A, Lengerich EJ (2011). The epidemiological association of altitude with chronic kidney disease: Evidence of protective effect.. Nephrology (Carlton).

[38] Kimmel PL, Miller G, Mendelson WB (1989). Sleep apnea syndrome in chronic renal disease.. Am J Med.

[39] Fletcher EC (1993). Obstructive sleep apnea and the kidney.. J Am Soc Nephrol.

[40] Hanly PJ, Pierratos A (2001). Improvement of sleep apnea in patients with chronic renal failure who undergo nocturnal hemodialysis.. N Engl J Med.

[41] Gusbeth-Tatomir P, Boisteanu D, Seica A, Buga C, Covic A (2007). Sleep disorders: a systematic review of an emerging major clinical issue in renal patients.. Int Urol Nephrol.

[42] Knight EL, Stampfer MJ, Rimm EB, Hankinson SE, Curhan GC (2003). Moderate alcohol intake and renal function decline in women: a prospective study.. Nephrol Dial Transplant.

[43] Menon V, Katz R, Mukamal K, Kestenbaum B, de Boer IH (2010). Alcohol consumption and kidney function decline in the elderly: alcohol and kidney disease.. Nephrol Dial Transplant.

[44] Schaeffner ES, Kurth T, de Jong PE, Glynn RJ, Buring JE (2005). Alcohol consumption and the risk of renal dysfunction in apparently healthy men.. Arch Intern Med.

[45] National Kidney Disease Education Program http://www.nkdep.nih.gov/.

[46] Wuerzner G, Pruijm M, Maillard M, Bovet P, Renaud C (2010). Marked association between obesity and glomerular hyperfiltration: a cross-sectional study in an African population.. Am J Kidney Dis.

[47] Ahmed SB, Fisher ND, Stevanovic R, Hollenberg NK (2005). Body mass index and angiotensin-dependent control of the renal circulation in healthy humans.. Hypertension.

